# Supplementary material for: 1,4-Naphthoquinone Motif in the Synthesis of New Thiopyrano[2,3-d]thiazoles as Potential Biologically Active Compounds
Source: Molecules. 2022 Nov 4;27(21):7575. doi: 10.3390/molecules27217575 (PMC9658586; doi:10.3390/molecules27217575)
Supplement: Supplementary file 1 [file molecules-27-07575-s001.zip › molecules-2006154-supplementary.pdf]

Supplementary information

Table of Contents

Copies of ^{1}H and ^{13}C NMR spectra of thiopyrano[2,3-*d*]thiazole derivatives S1-S12

NCI protocols for compounds **3.5** and **3.6** S13-S14

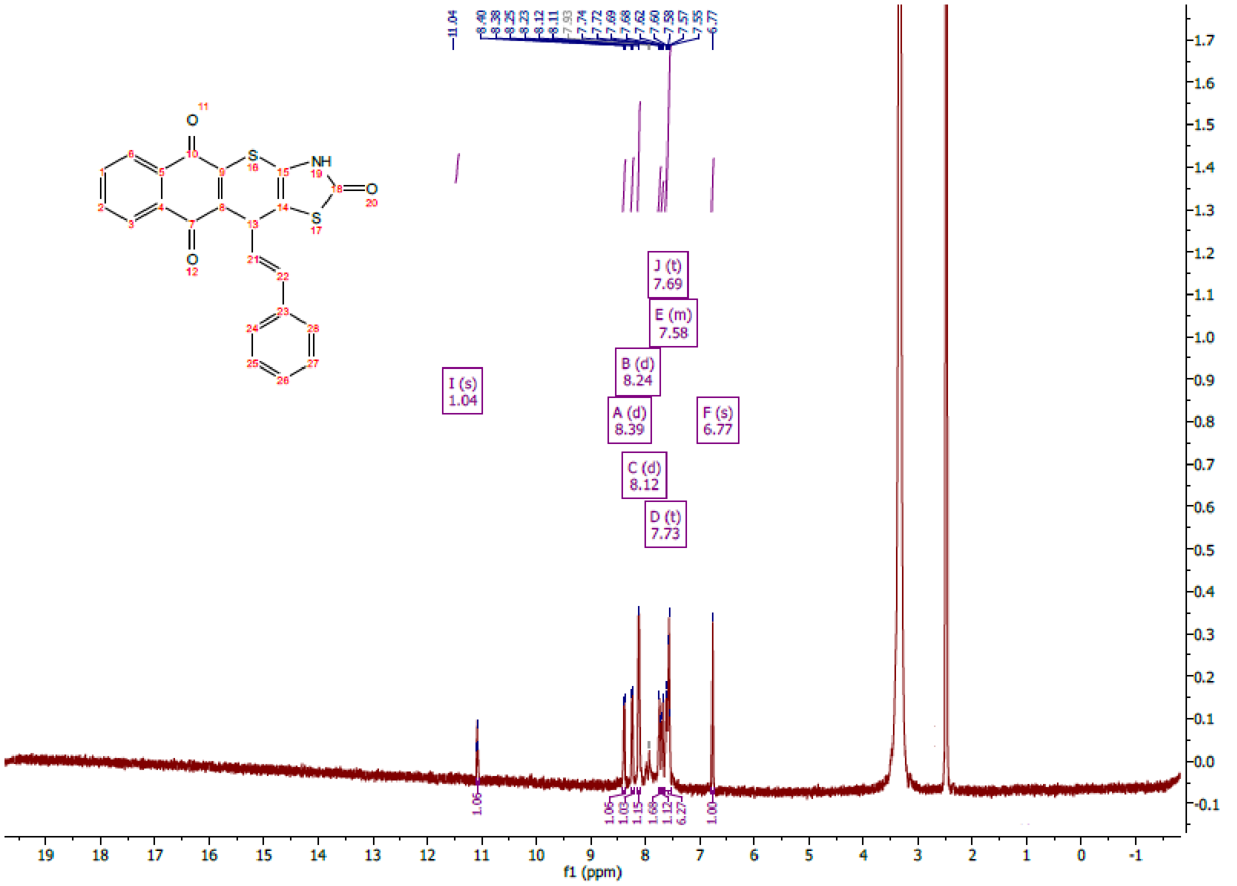

Figure S1. ^{1}H NMR Spectrum of compound **3.1**.

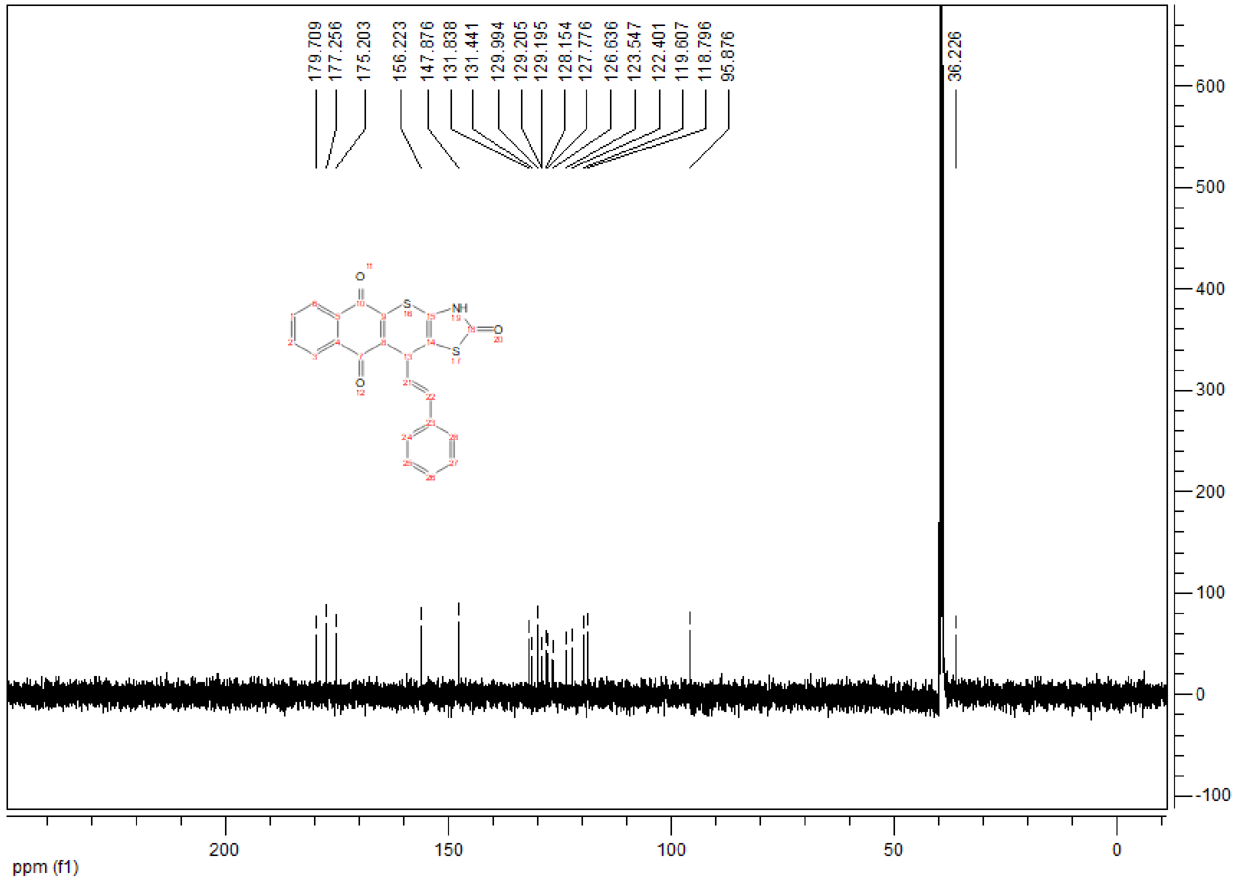

Figure S2. ^{13}C NMR Spectrum of compound **3.1**.

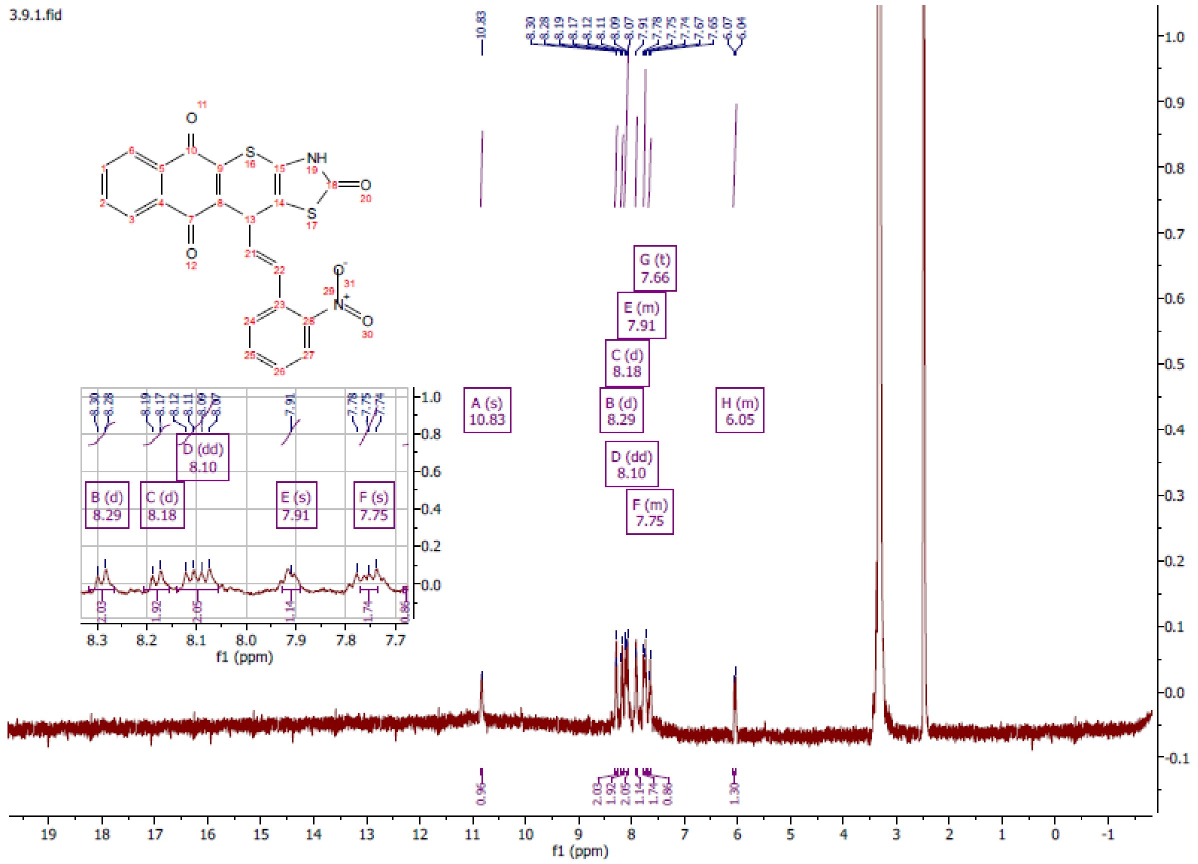

Figure S3. ^{1}H NMR Spectrum of compound **3.2**.

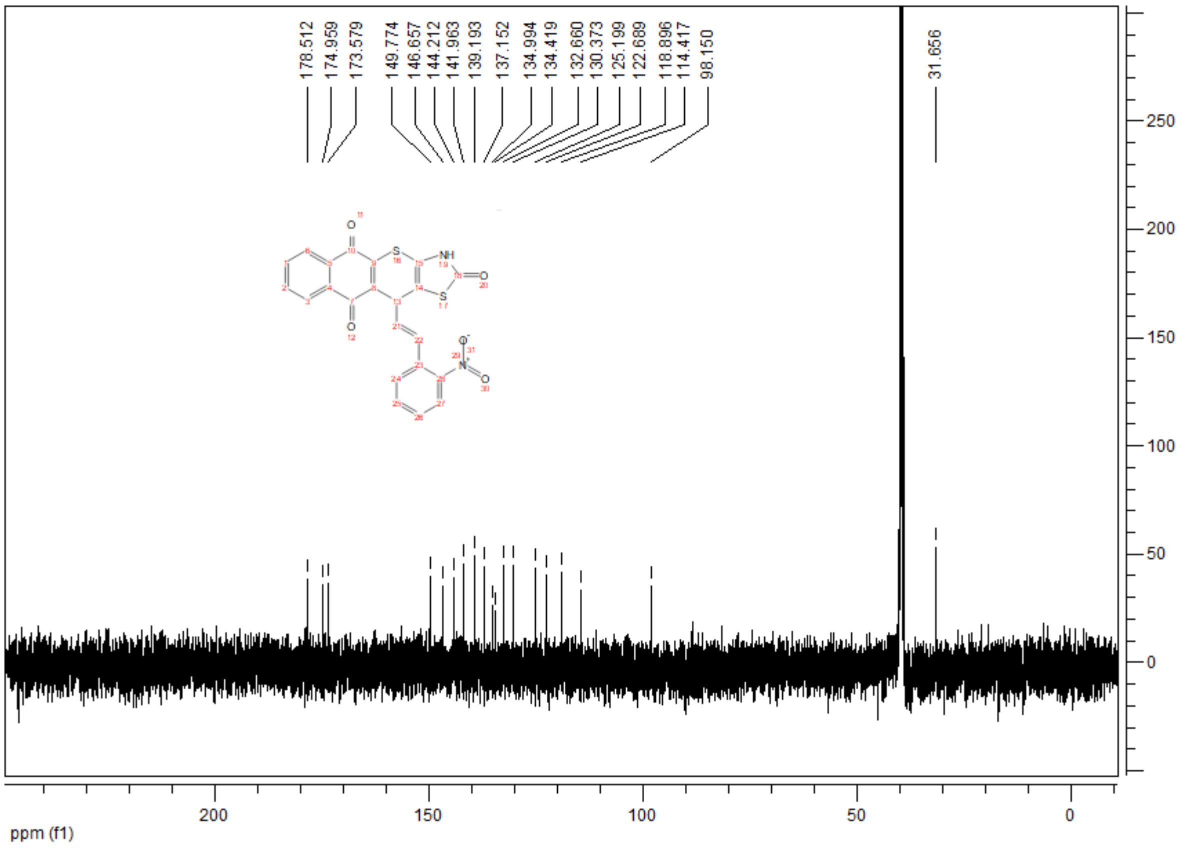

Figure S4. ^{13}C NMR Spectrum of compound **3.2**.

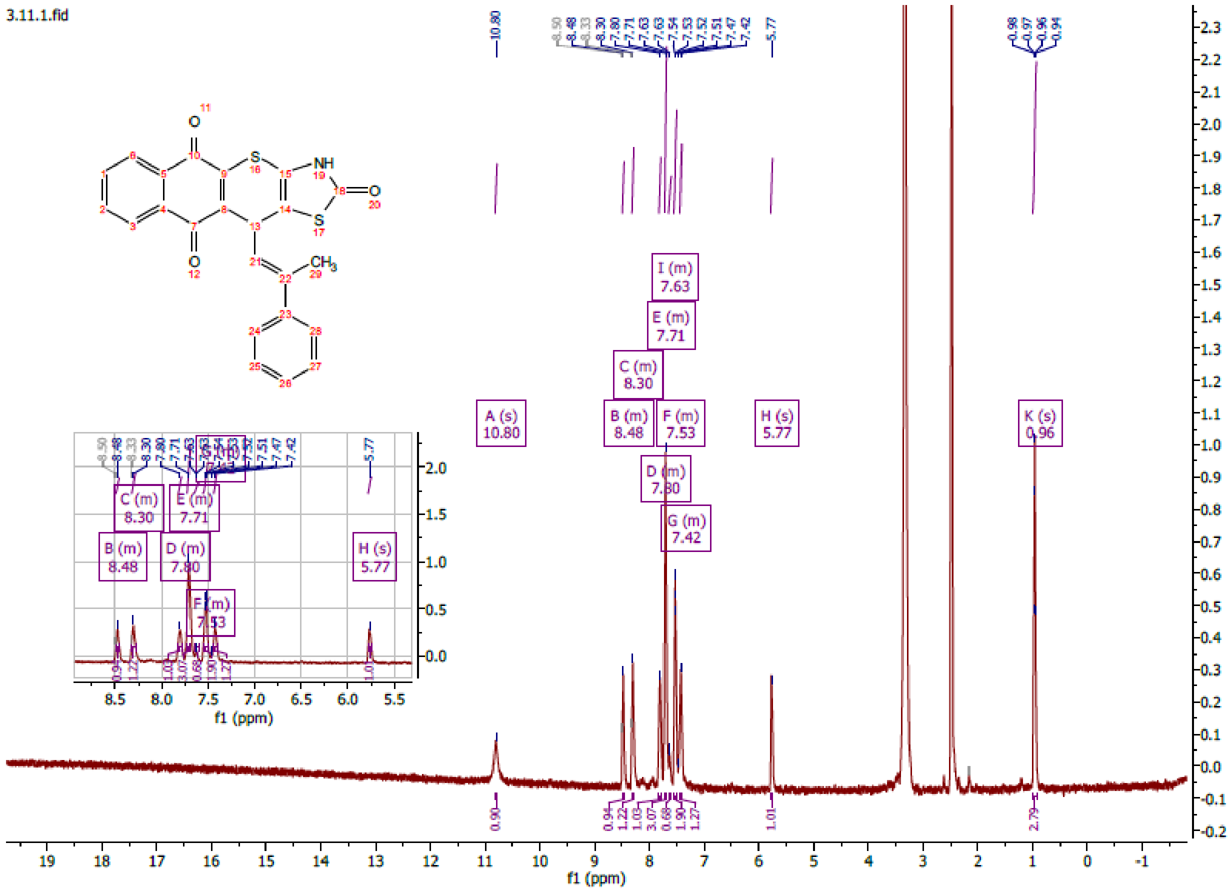

Figure S5 ^{1}H NMR Spectrum of compound **3.3**.

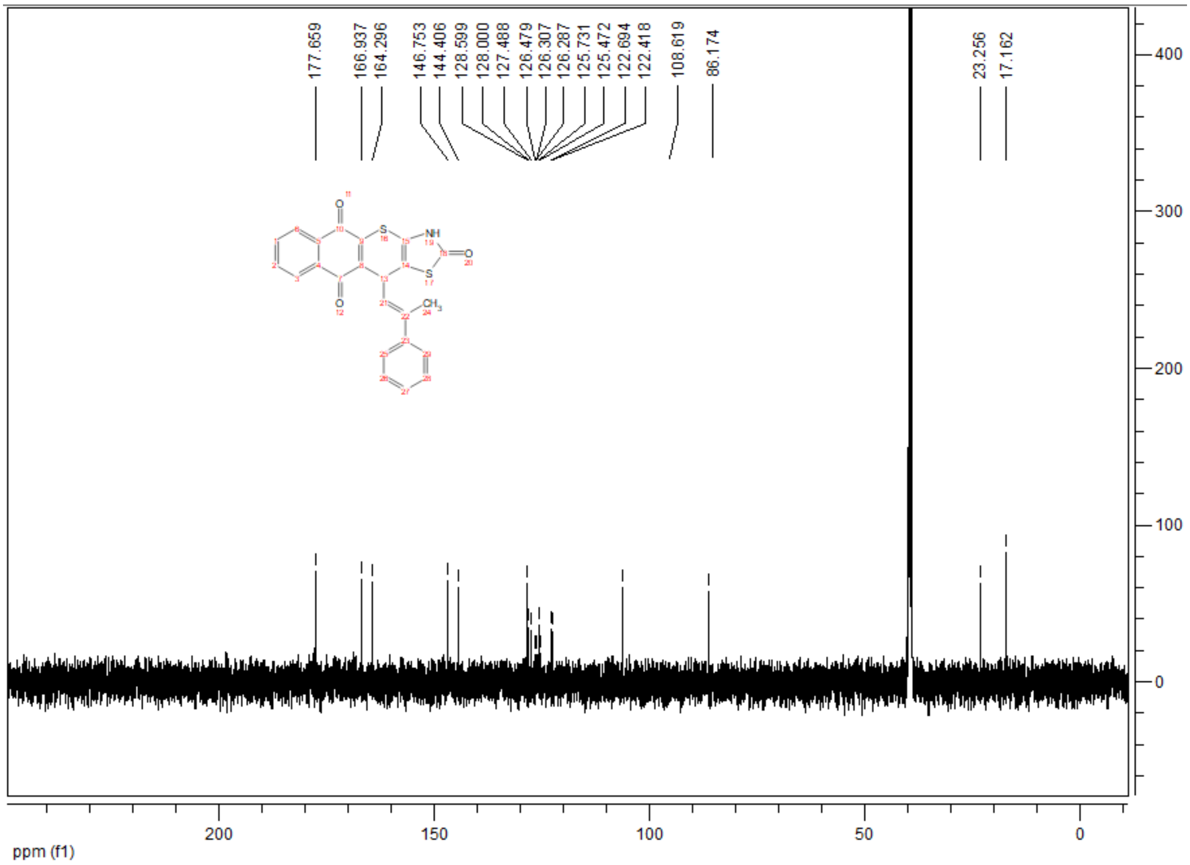

Figure S6. ^{13}C NMR Spectrum of compound **3.3**.

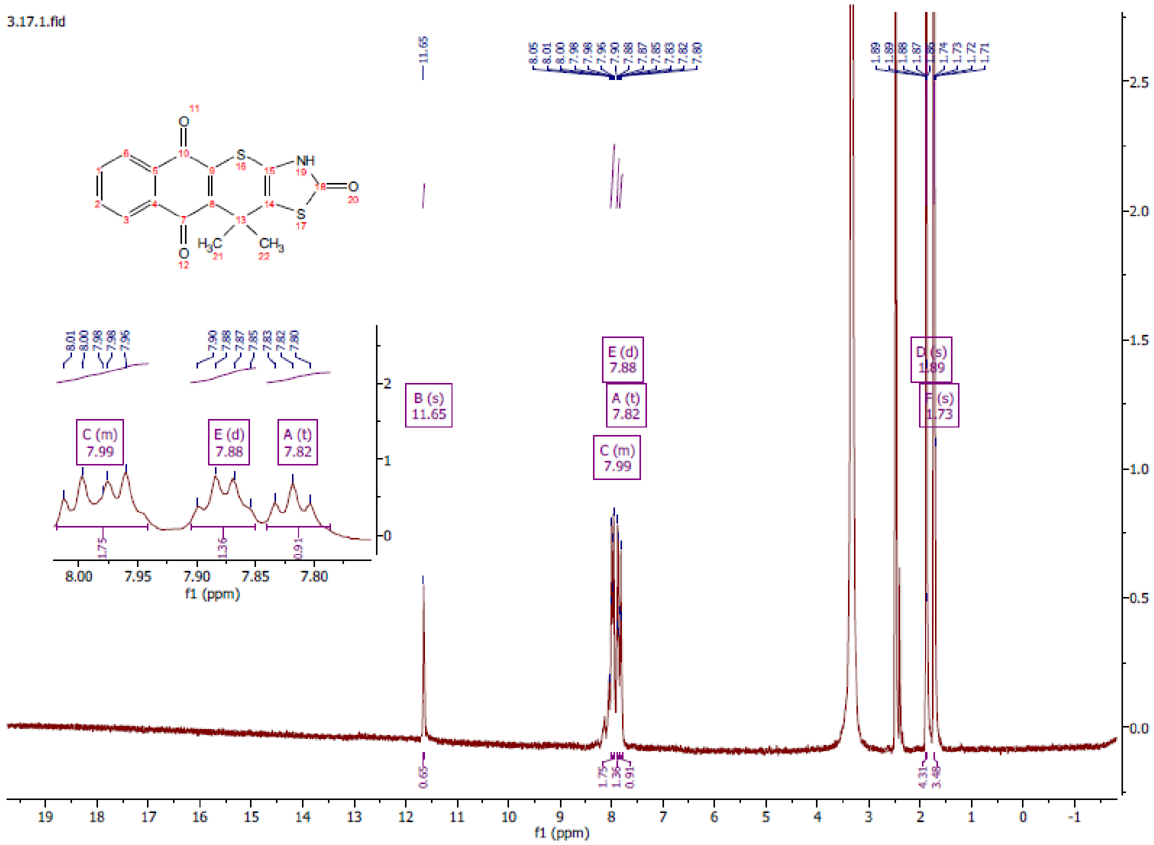

Figure S7. ^{1}H NMR Spectrum of compound **3.4**.

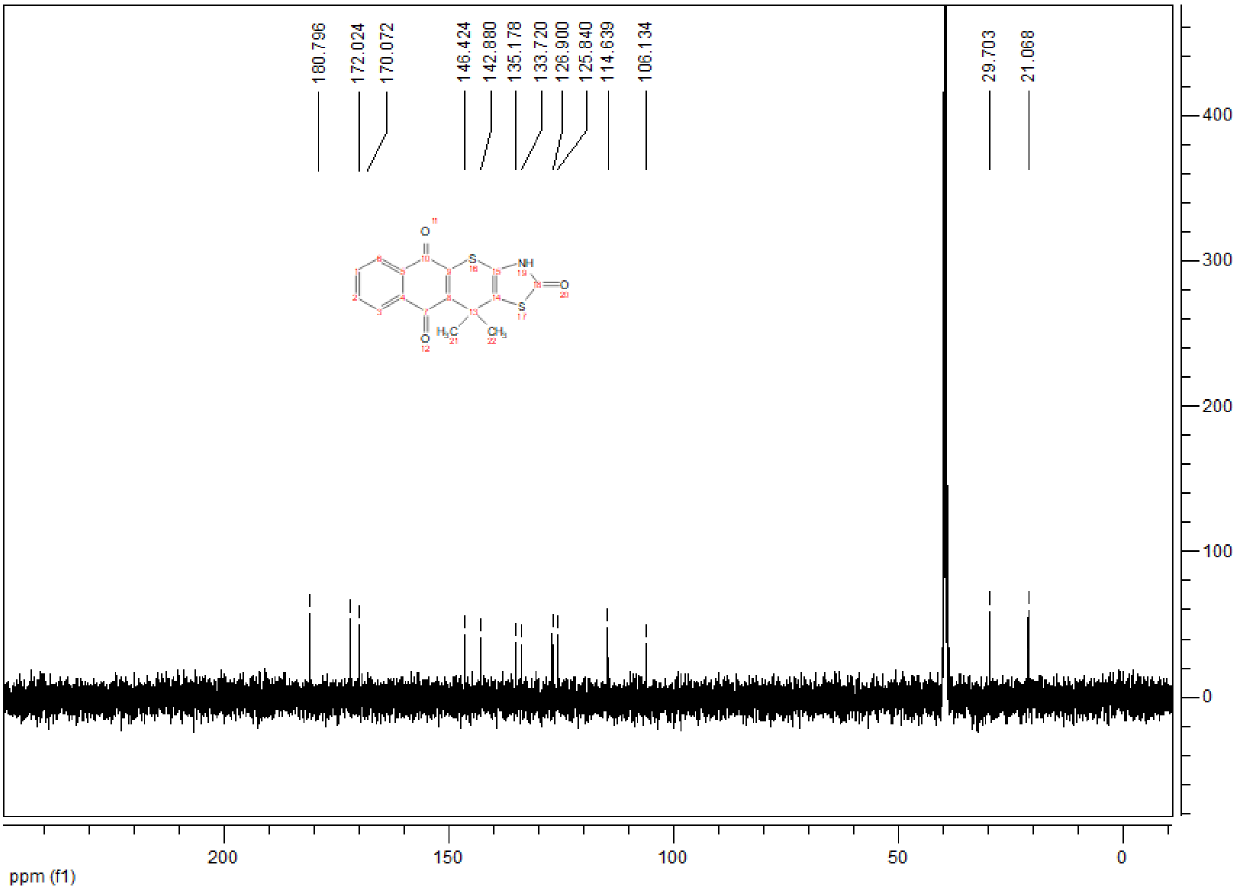

Figure S8. ^{13}C NMR Spectrum of compound **3.4**.

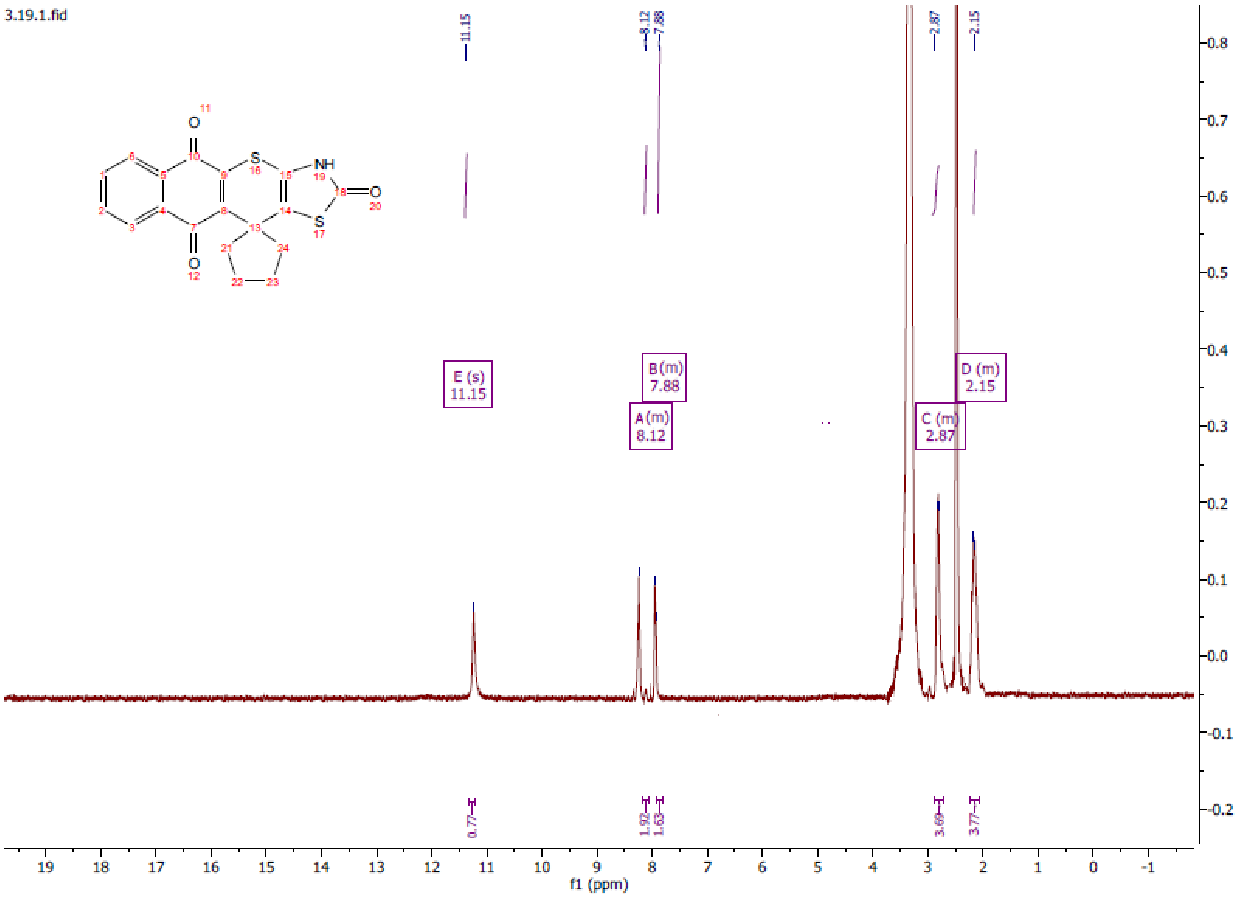

Figure S9. ^{1}H NMR Spectrum of compound **3.5**.

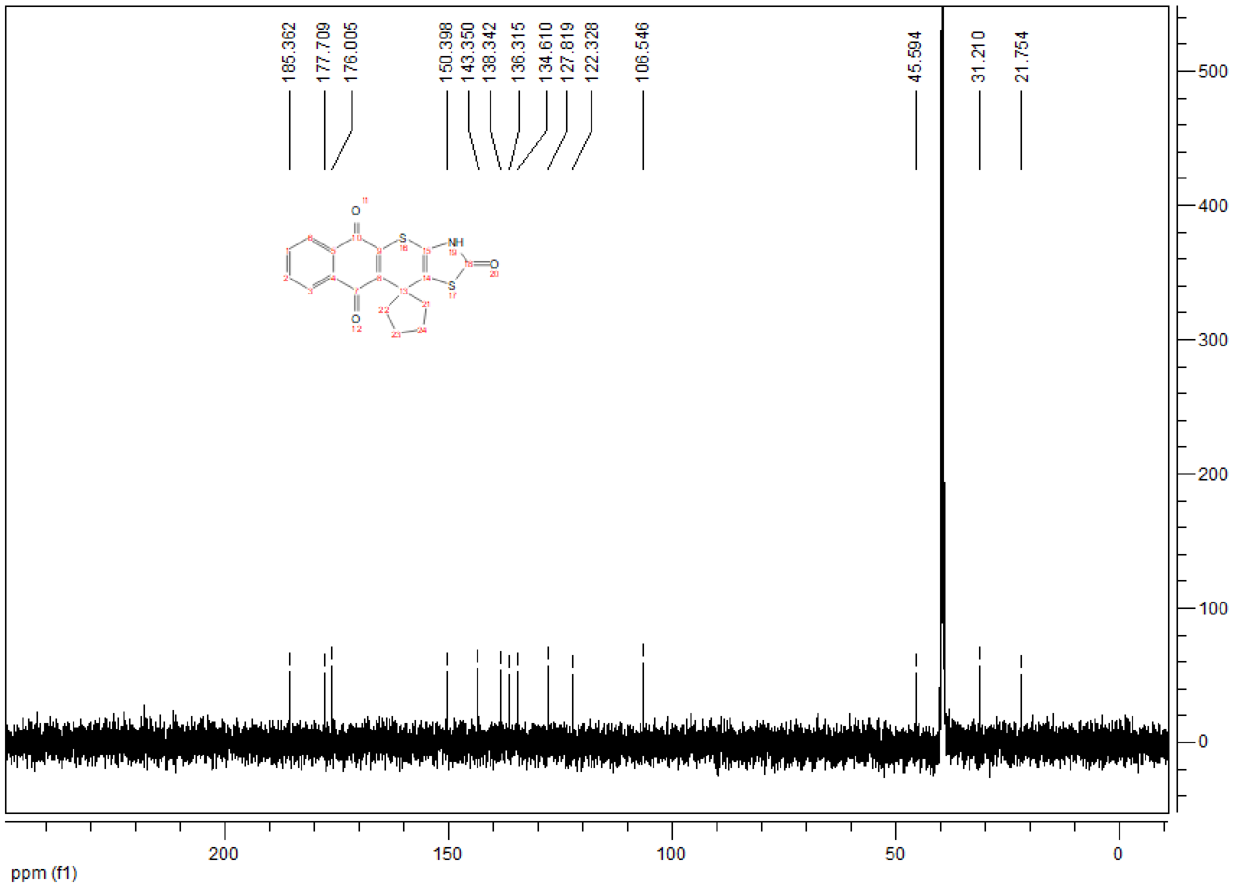

Figure S10. ^{13}C NMR Spectrum of compound **3.5**.

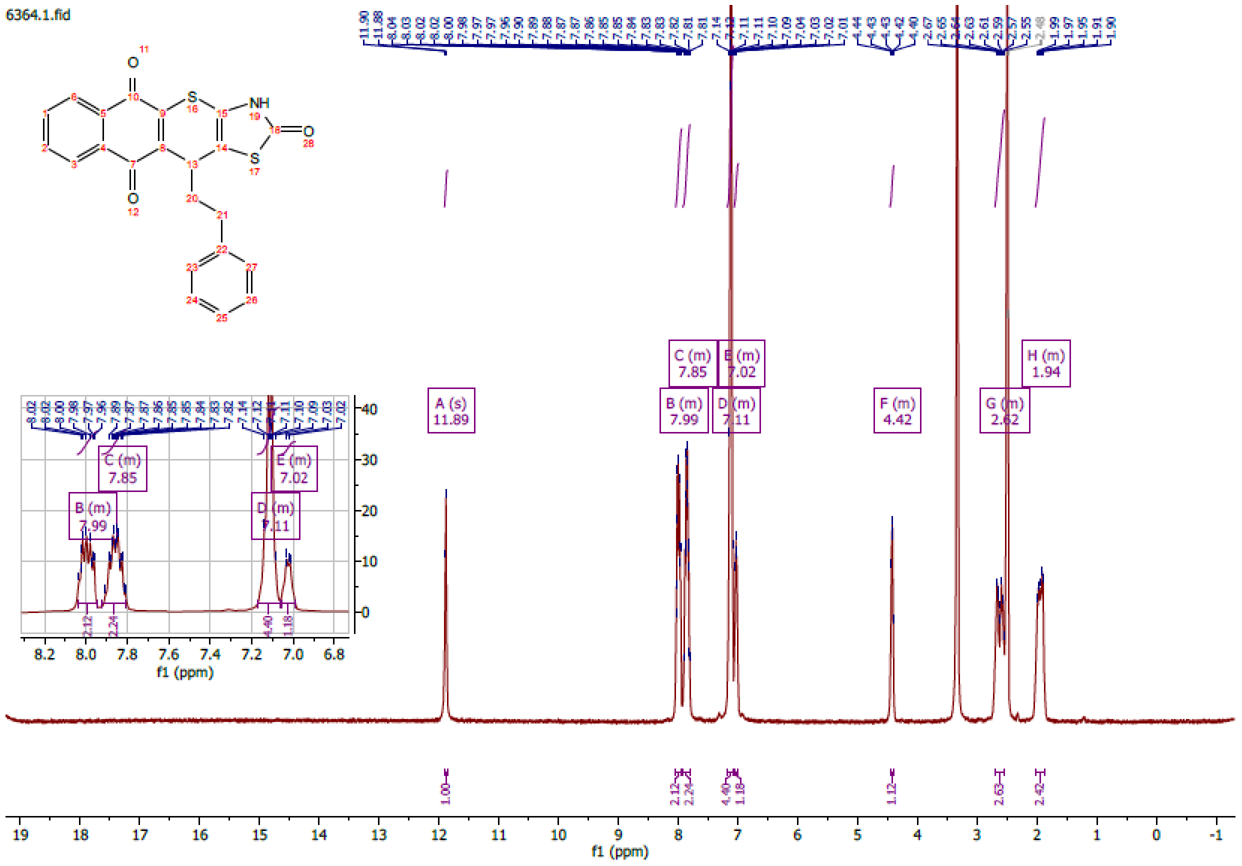

Figure S11. ^{1}H NMR Spectrum of compound **3.6**.

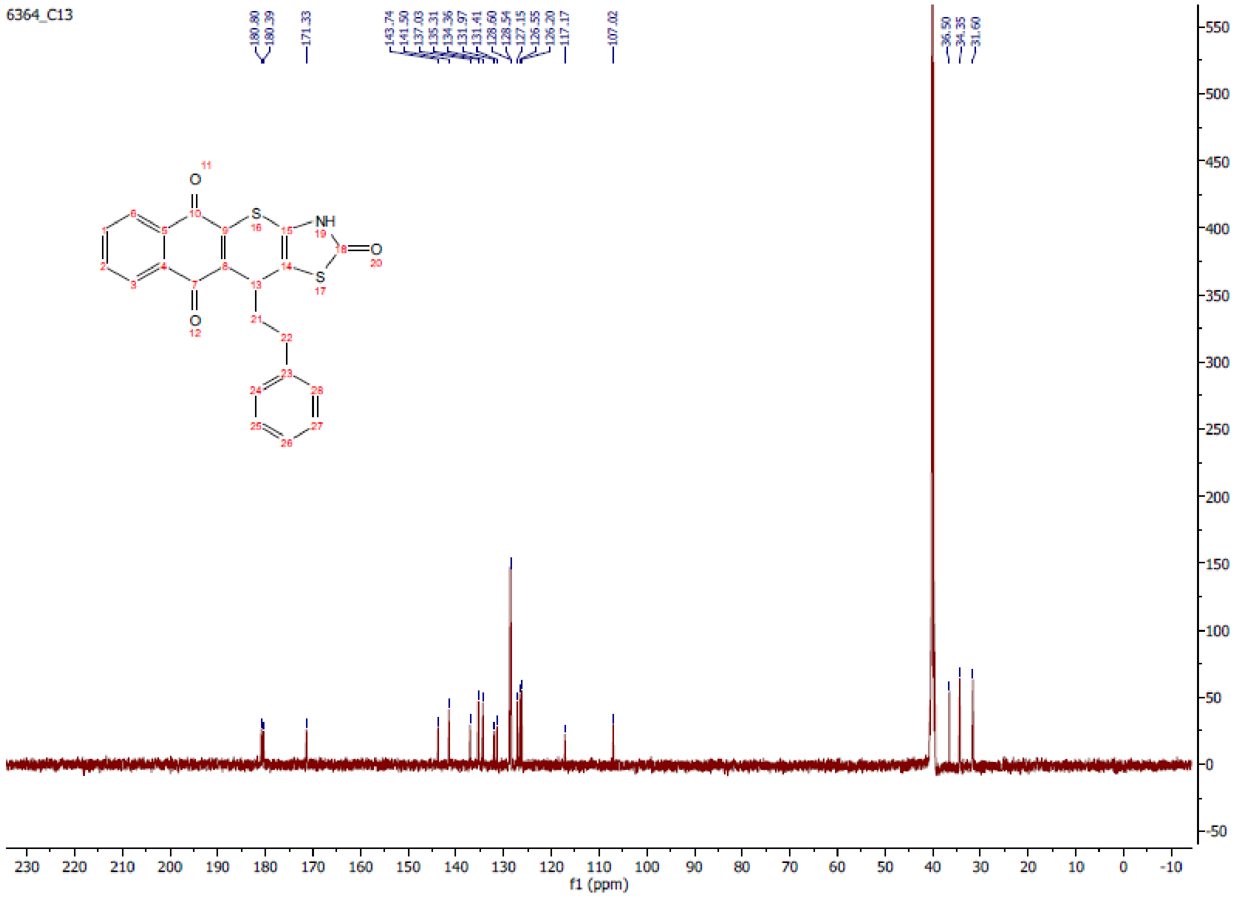

Figure S12. ^{13}C NMR Spectrum of compound **3.6**.

**Developmental Therapeutics Program**	**NSC:** D-748457 / 1	**Conc:** 1.00E-5 Molar	**Test Date:** Aug 25, 2008
**One Dose Mean Graph**	**Experiment ID:** 0808OS09		**Report Date:** Jun 17, 2022

Panel/Cell Line	Growth Percent
Leukemia	
CCRF-CEM	73.26
HL-60(TB)	89.60
K-562	70.86
MOLT-4	77.39
RPMI-8226	53.82
SR	59.89
Non-Small Cell Lung Cancer	
A549/ATCC	94.81
EKVX	54.98
HOP-62	91.86
HOP-92	81.81
NCI-H226	98.32
NCI-H23	87.20
NCI-H322M	87.71
NCI-H460	99.66
NCI-H522	78.24
Colon Cancer	
COLO 205	126.93
HCC-2998	104.46
HCT-116	93.07
HCT-15	98.45
HT29	110.84
KM12	97.41
SW-620	100.58
CNS Cancer	
SF-268	92.24
SF-295	91.86
SF-539	98.78
SNB-19	97.95
SNB-75	64.38
U251	101.84
Melanoma	
LOX IMVI	80.70
MALME-3M	98.88
M14	99.51
MDA-MB-435	113.31
SK-MEL-2	93.20
SK-MEL-28	107.79
SK-MEL-5	67.06
UACC-257	103.27
UACC-62	83.42
Ovarian Cancer	
IGROV1	55.15
OVCAR-3	91.11
OVCAR-4	83.80
OVCAR-5	105.14
OVCAR-8	83.94
NCI/ADR-RES	85.06
SK-OV-3	92.88
Renal Cancer	
786-0	96.94
A498	110.36
ACHN	83.21
CAKI-1	94.06
RXF 393	84.56
SN12C	92.33
TK-10	114.01
UO-31	55.54
Prostate Cancer	
DU-145	92.39
Breast Cancer	
MCF7	67.35
MDA-MB-231/ATCC	69.17
HS 578T	89.30
BT-549	98.67
T-47D	51.21
MDA-MB-468	89.71
Mean	88.33
Delta	37.12
Range	75.72

Mean Growth Percent - Growth Percent

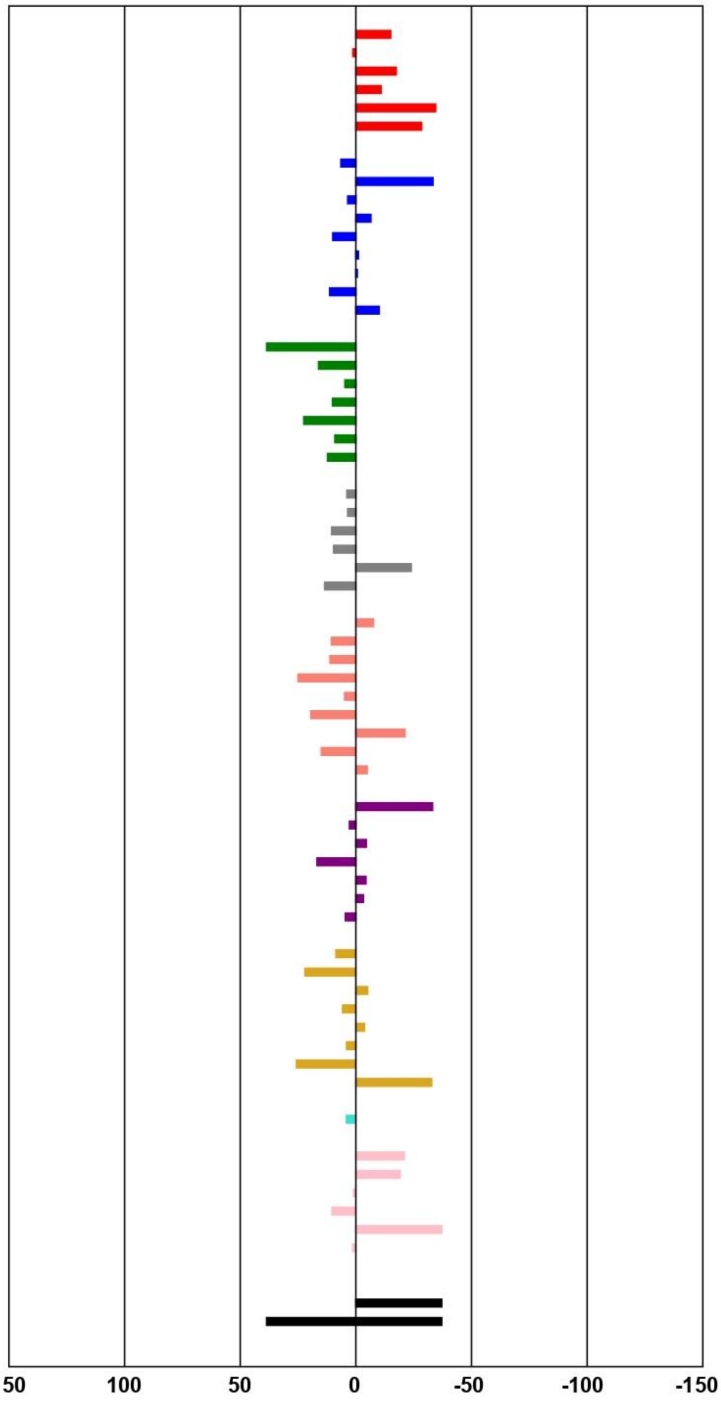

Figure S13. NCI-60 cell lines screening protocol in concentration 10 μM for compound **3.5**.

Developmental Therapeutics Program	NSC: D-831850 / 1	Conc: 1.00E-5 Molar	Test Date: Aug 02, 2021
One Dose Mean Graph	Experiment ID: 2108OS65		Report Date: Jun 19, 2022

Panel/Cell Line	Growth Percent
Leukemia	
CCRF-CEM	89.02
HL-60(TB)	71.67
K-562	82.89
MOLT-4	88.67
RPMI-8226	78.63
SR	74.66
Non-Small Cell Lung Cancer	
A549/ATCC	93.79
EKVX	84.87
HOP-62	115.83
HOP-92	80.03
NCI-H226	87.16
NCI-H322M	93.70
NCI-H460	87.18
NCI-H522	85.96
Colon Cancer	
COLO 205	113.14
HCC-2998	119.84
HCT-116	95.79
HCT-15	63.07
HT29	106.55
SW-620	106.32
CNS Cancer	
SF-268	96.65
SF-295	86.39
SF-539	99.71
SNB-19	98.97
SNB-75	130.11
U251	90.28
Melanoma	
LOX IMVI	57.17
MALME-3M	45.25
M14	84.60
MDA-MB-435	52.87
SK-MEL-2	89.25
SK-MEL-28	101.54
SK-MEL-5	65.46
UACC-257	83.60
UACC-62	80.61
Ovarian Cancer	
IGROV1	74.16
OVCAR-4	80.41
OVCAR-5	109.30
OVCAR-8	82.78
NCI/ADR-RES	90.53
SK-OV-3	94.62
Renal Cancer	
786-0	112.18
A498	109.69
ACHN	77.57
CAKI-1	93.53
RXF 393	98.74
SN12C	81.91
TK-10	214.67
UO-31	51.76
Prostate Cancer	
PC-3	79.17
DU-145	92.71
Breast Cancer	
MCF7	58.83
MDA-MB-231/ATCC	67.16
HS 578T	99.38
BT-549	125.25
T-47D	85.13
MDA-MB-468	102.22
Mean	90.58
Delta	45.33
Range	169.42

Mean Growth Percent - Growth Percent

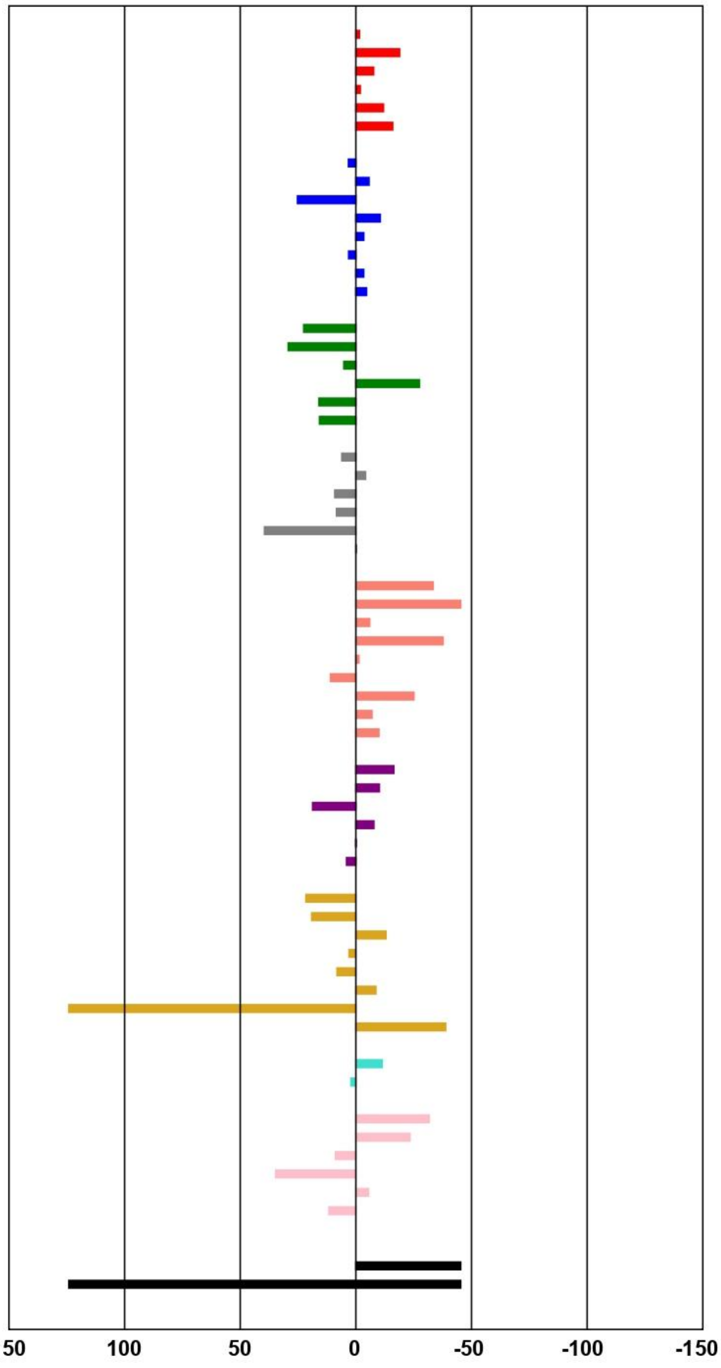

Figure S14. NCI-60 cell lines screening protocol in concentration 10 μM for compound **3.6**.
